# Global Invasion Potential and Niche Dynamics of *Phoracantha recurva* Newman, 1840 (Coleoptera: Cerambycidae) Under Climate Change

**DOI:** 10.3390/insects17070729

**Published:** 2026-07-15

**Authors:** Jiaqiang Zhao, Dongrui Sun, Huiru Wang, Te Liang, Keke Lan, Juan Shi

**Affiliations:** 1 Shijiazhuang Institute of Pomology, Hebei Academy of Agriculture and Forestry Sciences, Shijiazhuang 050061, China; jiaqiang_zhao@126.com; 2 Hebei Xiongan New Area City Ecosystem Observation and Research Station, Baoding 071703, China; sundr01@163.com (D.S.); liangte0705@126.com (T.L.); lankeke07@163.com (K.L.); 3 College of Forestry, Beijing Forestry University, Beijing 100083, China; 4 School of Science, Beijing Forestry University, Beijing 100083, China; whr2019@bjfu.edu.cn

**Keywords:** invasive forest pests, MaxEnt, *Eucalyptus* plantations, range expansion, COUE framework, biological invasion, climatic suitability

## Abstract

*Phoracantha recurva* Newman (Coleoptera: Cerambycidae), a wood-boring beetle native to Australia, threatens *Eucalyptus* L’Hér plantations worldwide. Despite established populations across North America, South America, Europe, and Africa, its global colonization potential and niche dynamics under climate change have received limited attention. Here, we used the maximum entropy (MaxEnt) model, integrating 844 occurrence records and eight bioclimatic variables, to predict the potential geographical distribution of *P. recurva* under current conditions and three future climate scenarios (SSP1-2.6, SSP3-7.0, and SSP5-8.5) for 2041–2070 and 2071–2100. We quantified niche dynamics using the COUE framework through calculations of niche stability, expansion, unfilling, and Schoener’s *D*, complemented by niche equivalency and similarity tests. The model exhibited excellent predictive performance (AUC = 0.954 ± 0.004; Boyce index = 0.999). Suitable habitats for *P. recurva* occurred primarily between 60° N and 55° S. Under all three future scenarios, total suitable area displayed a gradual decline while the potential distribution shifted toward higher latitudes, particularly across Europe and China. Niche analysis confirmed substantial niche conservatism during global invasion, yet revealed considerable niche unfilling during colonization of the Eurasian continent—indicating persistent potential for further range expansion. Niche comparisons between native and invaded ranges under model-projected distributions yielded expanded overlap across all regions except Africa, where invasive populations may undergo niche differentiation under changing climatic conditions. Our findings emphasize the ongoing invasion risk that *P. recurva* poses to major *Eucalyptus*-planting countries and call for strengthened inspection and quarantine measures to protect global forestry plantations.

## Simple Summary

*Phoracantha recurva* Newman, a wood-boring beetle native to Australia, inflicts substantial damage on *Eucalyptus* L’Hér plantations across multiple continents. We used species distribution modeling to predict climatically suitable areas for this pest under current and future climate conditions, and examined shifts in its environmental preferences during invasion. Suitable habitats span 60° N to 55° S. Future warming is projected to reduce total suitable area while shifting the distribution toward higher latitudes. Niche analysis revealed that *P. recurva* maintains its ancestral environmental preferences during global spread, yet has not fully occupied all suitable environments in Eurasia—suggesting continued potential for range expansion and informing targeted management strategies.

## 1. Introduction

*Eucalyptus* L’Hér is among the most widely cultivated fast-growing tree genera worldwide because of its rapid growth, broad environmental adaptability, and high commercial value [1]. Since its introduction from Australia during the nineteenth century, Eucalyptus has been established in more than 100 countries and regions [2]. Brazil, China, and India possess the largest plantation areas, collectively producing approximately 250 million cubic meters of timber annually, equivalent to nearly 37% of global plantation wood production [3]. In China, eucalyptus achieves timber production efficiencies 1.78, 2.27, and 3.57 times those of *Populus* L. spp., *Cunninghamia lanceolata* (Lamb.) Hook., and *Pinus massoniana* Lamb., respectively [4].

*Phoracantha recurva* Newman (Coleoptera: Cerambycidae) is a cerambycid native to Australia [5]. According to the Centre for Agriculture and Bioscience International (CABI), this species has established populations in more than 20 countries and has caused economic losses in the United States, Brazil, Portugal, and several other regions [6]. In Zambia, drought stress combined with infestations by *Phoracantha* species caused mortality rates of 34% in five-year-old *Eucalyptus grandis* W. Hill ex Maiden and 29% in six-year-old *E. cloeziana* F. Muell plantations [7]. Together with *P. semipunctata* Fabricius, *P. recurva* has become a major cause of *Eucalyptus* decline and mortality in California and other Mediterranean-climate regions [8]. Given its economic importance, the species has been listed as an A2 quarantine pest by both the European and Mediterranean Plant Protection Organization (EPPO) and the North American Plant Protection Organization (NAPPO).

Species distribution models (SDMs) integrate species occurrence data with environmental variables to characterize habitat suitability and predict potential distributions [9]. Among the available SDM approaches, the maximum entropy (MaxEnt) algorithm has become one of the most widely applied tools because of its strong predictive performance and robustness [10]. Compared with other modeling approaches, such as generalized boosted models (GBMs) and generalized additive models (GAMs), MaxEnt is less sensitive to sample sizes and produces stronger predictions when occurrence records are scarce [11]. Moreover, because MaxEnt relies solely on presence records and environmental predictors, it avoids uncertainties associated with absence data [12]. Consequently, MaxEnt has been extensively used to assess invasion risk, identify climatically suitable habitats, and support conservation planning [13,14,15].

Previous studies have demonstrated that countries with extensive *Eucalyptus* industries, including China, India, and Portugal, provide highly suitable environments for *P. semipunctata*, highlighting the vulnerability of these regions to invasive *Phoracantha* species [16]. Compared with *P. semipunctata*, *P. recurva* exhibits greater fecundity and a shorter life cycle, traits that may enhance its invasion success [17]. Nevertheless, information on the global invasion potential and climatic niche dynamics of *P. recurva* remains limited. In particular, whether this species conserves its ancestral climatic niche during invasion or undergoes niche shifts in newly colonized regions remains unclear.

To address these knowledge gaps, we used an optimized MaxEnt model to predict the current and future potential distribution of *P. recurva* under multiple climate-change scenarios. We then quantified niche dynamics between native and invaded populations by estimating niche stability, expansion, and unfilling, together with Schoener’s *D* and niche equivalency and similarity tests. Specifically, we aimed to: (i) identify the principal climatic factors governing the global distribution of *P. recurva*; (ii) assess changes in climatically suitable areas under future climate scenarios; and (iii) determine whether climatic niche conservatism or niche shifts characterize the invasion process of this species.

## 2. Materials and Methods

### 2.1. Occurrence Data

**Figure 1:**
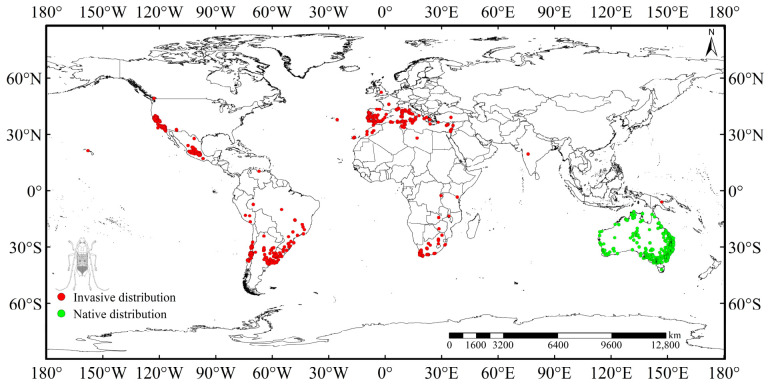
Occurrence records of *P. recurva*. Two different-colored dots indicate distribution records.

Occurrence records of *Phoracantha recurva* were compiled from several publicly available databases, including the Global Biodiversity Information Facility (GBIF Occurrence Download. Available online: https://doi.org/10.15468/dl.5855qe, accessed on 18 April 2025), the Centre for Agriculture and Bioscience International (CABI, https://www.cabi.org/, accessed on 18 April 2025), the Barcode of Life Data System (BOLD, https://v4.boldsystems.org/, accessed on 18 April 2025), and the Atlas of Living Australia (ALA, https://www.ala.org.au/, accessed on 18 April 2025). Additional records were obtained through an extensive review of published literature [5,18]. To reduce spatial sampling bias and spatial autocorrelation, occurrence records separated by less than 5 km were spatially filtered using the spThin package (version 0.2.0) in R version 4.4.1. Only one occurrence point was retained within each 2.5 arcmin grid cell [19]. After data cleaning and thinning, 844 occurrence records were retained for subsequent analyses. These records were distributed mainly across North America, Europe, and Africa (Figure 1).

### 2.2. Bioclimatic Variables

Nineteen bioclimatic variables (BIO1–BIO19) known to influence species distributions were downloaded from the CHELSA v2.1 database (https://chelsa-climate.org/) for the period 1981–2010 (Table S1). Future climate projections were used for three Shared Socioeconomic Pathway scenarios (SSP1-2.6, SSP3-7.0, and SSP5-8.5) for two future periods: 2041–2070 and 2071–2100. To evaluate the effects of climate change under CMIP6 scenarios on the potential distribution of *P. recurva*, we used the ensemble mean across five general circulation models (GCMs): GFDL-ESM4, IPSL-CM6A-LR, MPI-ESM1-2-HR, MRI-ESM2-0, and UKESM1-0-LL. All climatic variables were resampled to a spatial resolution of 2.5 arcmin using the bilinear interpolation method in ArcGIS 10.8.

To minimize multicollinearity among predictor variables, Pearson correlation analyses were conducted in R 4.4.1 using values extracted at occurrence locations (Figure S1). For variable pairs exhibiting strong correlations (|r| > 0.7), only the variable with the greater ecological relevance and higher contribution to preliminary model performance was retained [20]. Eight variables were ultimately selected for *P. recurva* (Figure S2A).

### 2.3. MaxEnt Model Optimization

In the MaxEnt model, the regularization multiplier (RM) controls model complexity through constraint placement, while feature combinations (FC) penalize additional constraints (e.g., variables) [21]. Appropriate optimization of these parameters is essential to avoid model overfitting and improve predictive performance. The ENMeval package was used to optimize model settings by evaluating eight feature class combinations (L, LQ, LQH, LQHP, LQHPT, QHP, QHPT, and HPT) in combination with eight regularization multipliers (0.5–4.0, at intervals of 0.5). The parameter combination producing the lowest ΔAICc value was considered optimal. The optimized settings were FC = QHP and RM = 1.5 (Figure S2B). For model calibration, 75% of the occurrence records were randomly assigned to the training dataset, whereas the remaining 25% were reserved for model testing. Ten replicate runs were performed using identical parameter settings, and the average prediction across all replicates was used as the final output.

To further explore climatic suitability within invaded regions and assess the invasion status of established populations, regional-scale models were additionally constructed. Stable invaded populations were identified according to the framework proposed by Gallien et al. [22]. Detailed parameter settings and optimization procedures for regional models were provided in the Supplementary Materials (Table S2; Figures S3 and S4).

### 2.4. Suitability Classification and Model Validation

Threshold selection should be objective, equitable, and discriminable [23]. Based on the current distribution of *P. recurva*, predicted suitability values were classified into four categories using the Jenks natural breaks method implemented in ArcGIS 10.8: unsuitable, slightly suitable, moderately suitable, and highly suitable habitats.

Model performance was evaluated using both the area under the receiver operating characteristic curve (AUC) and the Boyce index (BI). The AUC is among the most widely used metrics for evaluating SDM performance [24]. AUC values range from 0 to 1, with values > 0.9 indicating excellent predictive ability [25]. We additionally calculated the Boyce index, which provides a presence-only evaluation of model performance [26]. BI values range from −1 to 1, with positive values indicating consistency between predicted suitability and observed species occurrences [27]. The BI was calculated with the ecospat package via a moving-window approach. The window width was 1/10 of the suitability range, and the resolution was 100 focals. Spearman analysis was selected as the correlation calculation method. Given that the Boyce index is less sensitive to the quantity and spatial configuration of background points [28], the combined use of AUC and BI provides a more comprehensive assessment of model reliability. In addition, omission rates were examined to evaluate potential model overfitting and spatial autocorrelation [29].

### 2.5. Niche Shift Analysis

#### 2.5.1. Niche Comparison Within the Observed Distribution Range

Niche dynamics between native and invaded populations of *P. recurva* were quantified using the COUE framework proposed by Broennimann et al. [30]. This framework is widely regarded as a robust approach for assessing climatic niche shifts during biological invasions. A 50 km buffer was generated around each occurrence record, and 10,000 random background points were sampled within the buffered areas to characterize available environmental conditions [31]. Environmental values extracted from occurrence and background points were summarized using principal component analysis (PCA), and the first two principal components were projected onto a 100 × 100 environmental grid. The kernel density function was applied to smooth the occurrence density of *P. recurva* within the environmental spaces of both the native and invaded ranges. Climatic niche dynamics were partitioned into three components: niche stability (environmental conditions occupied in both native and invaded ranges), niche expansion (conditions occupied exclusively in invaded ranges), and niche unfilling (conditions occupied only in native ranges) [32]. Values exceeding 0.1 were considered indicative of substantial niche expansion or unfilling [33,34].

Schoener’s *D* (Equation (1)) was used to quantify niche overlap, ranging from 0 (no overlap) to 1 (complete overlap):

(1) $$\begin{array}{c} D=1-\frac{1}{2}\left(\sum_{ij}\left|z_{1_{ij}}-{z_{2}}_{ij}\right|\right) \end{array}$$

where *z*_1*ij*_ and *z*_2*ij*_ denote the occurrence densities of the species in grid cell (*i, j*) within the native and invaded ranges, respectively. *D* values were interpreted as follows: 0–0.2, no or very limited overlap; 0.2–0.4, low overlap; 0.4–0.6, moderate overlap; 0.6–0.8, high overlap; and 0.8–1.0, very high overlap [35].

Niche equivalency and similarity tests were further performed to assess the degree of climatic niche divergence between native and invaded populations. Both tests were repeated 1000 times to generate null distributions of Schoener’s *D* values. Rejection of the null hypothesis in the niche equivalency test indicates that native and invaded populations occupy non-identical climatic niches. Rejection of the null hypothesis in the niche similarity test suggests that the climatic conditions occupied in the invaded range are more similar to those in the native range than expected under random environmental occupancy [36].

#### 2.5.2. Niche Comparison Within the Model-Predicted Spatial Distribution

The Hellinger distance provides an ecologically meaningful metric for comparing model predictions [37]. Warren et al. transformed this measure and recommended its combined use with Schoener’s *D* to evaluate ecological niche overlap among SDM predictions:

(2) $$\begin{array}{c} I\left(P_{x},P_{y}\right)=1-\frac{1}{2}{\left(\sqrt{\sum_{i}{\left(\sqrt{P_{x,i}}-\sqrt{P_{y,i}}\right)}^{2}}\right)}^{2} \end{array}$$

where *P_x,i_* and *P_y,i_* represent the SDM-predicted probability of species occurrence in grid cell *i* for the native and invaded ranges, respectively. The modified *I*(*P_x_, P_y_*) ranges from 0 to 1, ensuring comparability with other niche overlap measures [35]. This metric was calculated using ENMTools 1.4.4.

## 3. Results

### 3.1. Model Performance and Accuracy

**Figure 2:**
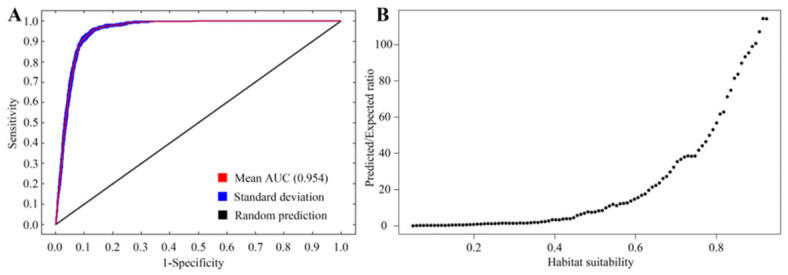
Accuracy evaluation of the MaxEnt model for *P. recurva*. (**A**) ROC curve of MaxEnt for *P. recurva*. (**B**) Predicted-to-expected ratio versus habitat suitability plot.

A total of 844 occurrence records and eight bioclimatic variables were used to predict the potential distribution of *P. recurva*. The optimized MaxEnt model exhibited excellent predictive performance. Across ten replicate runs, the mean AUC value reached 0.954 ± 0.004, while the Boyce index was 0.999, indicating strong agreement between model predictions and observed occurrence records (Figure 2). Regional-scale models also performed well. All regional models produced AUC and Boyce index values exceeding 0.90, except for the African model, which yielded a Boyce index of 0.894 (Figure S5). Omission rates for the test samples closely matched predicted rates (Figures S6 and S7), confirming the absence of spatial autocorrelation in the modeling data. Overall, these results demonstrate that the optimized MaxEnt model reliably captured the climatic requirements of *P. recurva* and was suitable for predicting its potential distribution at both global and regional scales.

### 3.2. Potential Geographical Distribution of P. recurva Under Different Climate Conditions

#### 3.2.1. Potential Geographical Distribution Under Near-Current Climate Conditions

**Figure 3:**
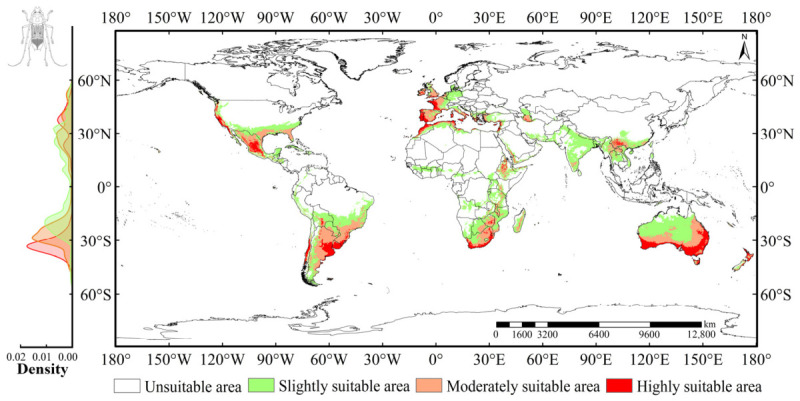
Global potential distribution of *P. recurva* under near current climate conditions.

**Figure 4:**
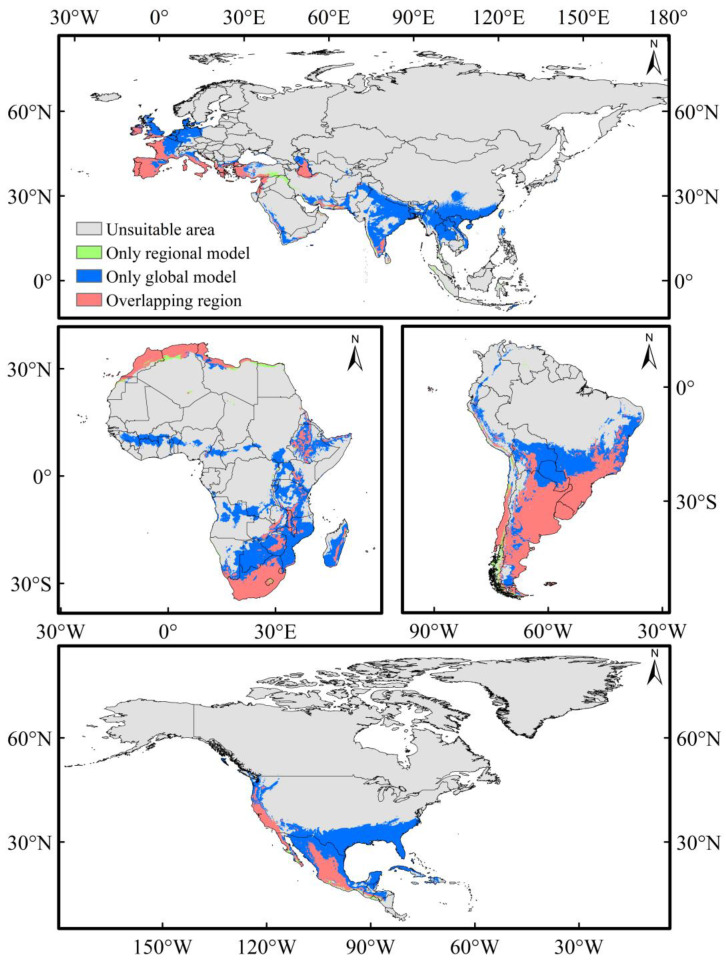
The regional and global models of *P. recurva*.

Under near-current climate conditions, suitable habitats for *P. recurva* occurred primarily between 60° N and 55° S, encompassing Mediterranean, temperate oceanic, and subtropical monsoon climate zones (Figure 3). Slightly, moderately, and highly suitable areas covered 1269.61 × 10^4^ km^2^, 669.40 × 10^4^ km^2^, and 305.94 × 10^4^ km^2^, respectively. Highly suitable areas were concentrated mainly in southeastern South America, the western Gulf Coast of North America, and the western Iberian Peninsula, and many of these regions already support stabilizing populations of *P. recurva* (Figure 4). In China, suitable habitats were distributed predominantly in Yunnan, Sichuan, and Guizhou provinces, together with adjacent southwestern regions.

#### 3.2.2. Potential Geographical Distribution Under Future Climate Conditions

**Figure 5:**
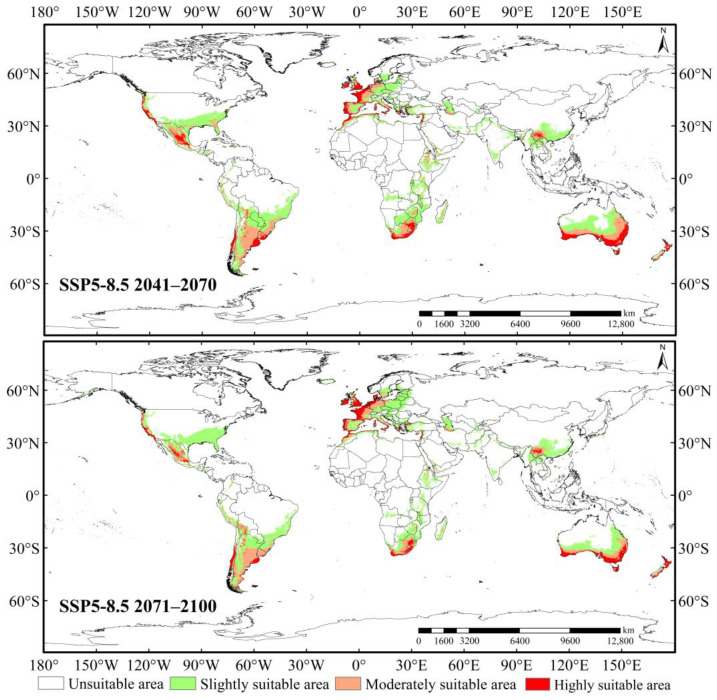
The predicted geographical distribution of *P. recurva* under SSP5-8.5.

Under future climate scenarios (SSP1-2.6, SSP3-7.0, and SSP5-8.5), substantial changes in habitat suitability were projected across South Asia, the Gulf of Mexico region, and Europe (Figure 5 and Figure S8). The Indian Peninsula exhibited particularly pronounced contractions in suitable habitat. Extensive areas previously classified as slightly suitable became increasingly restricted to southwestern India. Under the high-emission SSP5-8.5 scenario, most slightly suitable habitats in central India disappeared by the end of the century. Marked changes were also predicted for the Gulf of Mexico region. Moderately suitable habitats near the Mississippi River Plain persisted only during the 2070s under SSP1-2.6. Under all other scenario–period combinations, extensive moderately suitable areas in the northern Gulf Coast contracted substantially and were largely replaced by slightly suitable habitats. In China, the potential distribution expanded northward under future climates. Under SSP5-8.5, newly suitable areas extended as far north as Zhejiang and Jiangsu provinces. A comparable pattern was observed in Europe, where the potential range shifted northward and eastward. Parts of the Scandinavian Peninsula, Czechia, and Poland gradually became climatically suitable, while moderately to highly suitable habitats emerged in Germany and Denmark. Several major *Eucalyptus*-growing regions, including the Wogera districts of Ethiopia, Uruguay, and the Brazilian states of Paraná and Rio Grande do Sul, remained moderately or highly suitable under future climates. These regions may therefore face an elevated risk of colonization by *P. recurva*.

**Figure 6:**
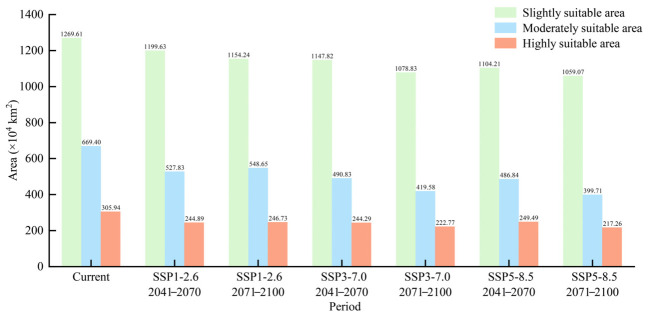
Areas of the potential geographic distribution of *P. recurva* under near current condition and SSP1-2.6, SSP3-7.0, and SSP5-8.5.

Total suitable area declined across all three scenarios (Figure 6). SSP5-8.5 produced the most pronounced reductions, with slightly, moderately, and highly suitable areas reaching minima of 1059.07 × 10^4^ km^2^, 399.71 × 10^4^ km^2^, and 217.26 × 10^4^ km^2^. Relative to near-current conditions, these represent reductions of 16.58%, 40.29%, and 28.99%, respectively.

#### 3.2.3. Shifts in the Potential Distribution of *P. recurva* Under Climate Change

Across all three climate scenarios, *P. recurva* exhibited broadly consistent distributional shifts (Figure S8). Suitable areas expanded in northern and eastern Europe, the northeastern Gulf of Mexico, western South America, and parts of southern China, reflecting a pronounced poleward displacement of the species’ potential range. Europe was projected to become increasingly favorable for establishment under future climates. By contrast, climatically suitable areas contracted across South Asia, Southeast Asia, central South America, and Australia. Because these regions are concentrated primarily between 30° N and 30° S, the observed contractions indicate a general decline in habitat suitability throughout tropical and subtropical regions.

### 3.3. Relationship Between Potential Distribution and Bioclimatic Variables

**Figure 7:**
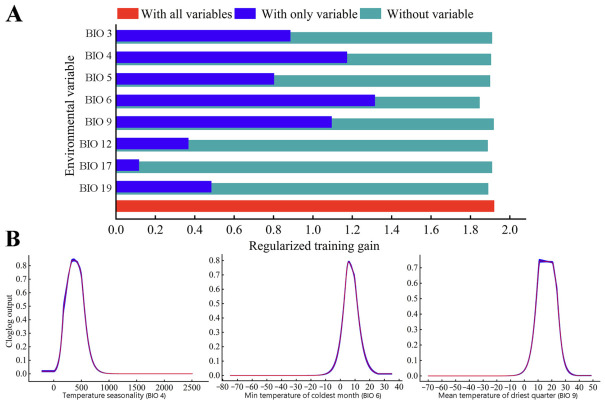
(**A**) Importance of climate variables in predicting the distribution of *P. recurva*. (**B**) The response curves of *P. recurva*. Red indicates the mean response from 10 replicate Maxent runs; blue indicates mean ± SD.

Jackknife analysis identified the bioclimatic variables most strongly influencing *P. recurva* distribution. Minimum temperature of the coldest month (BIO6), mean temperature of the driest quarter (BIO9), and temperature seasonality (BIO4) emerged as the dominant drivers (Figure 7A). Temperature variables thus constituted the key factors governing the species’ distribution.

Response curves revealed similar influences for all three top-ranked variables, with suitability rising and then declining as each variable increased (Figure 7B). Optimal suitability occurred when BIO6 ranged from 2.69 to 11.16 °C, BIO9 from 8.98 to 23.94 °C, and BIO4 from 193.40 to 544.59.

### 3.4. Analysis of Niche Dynamics in P. recurva

**Figure 8:**
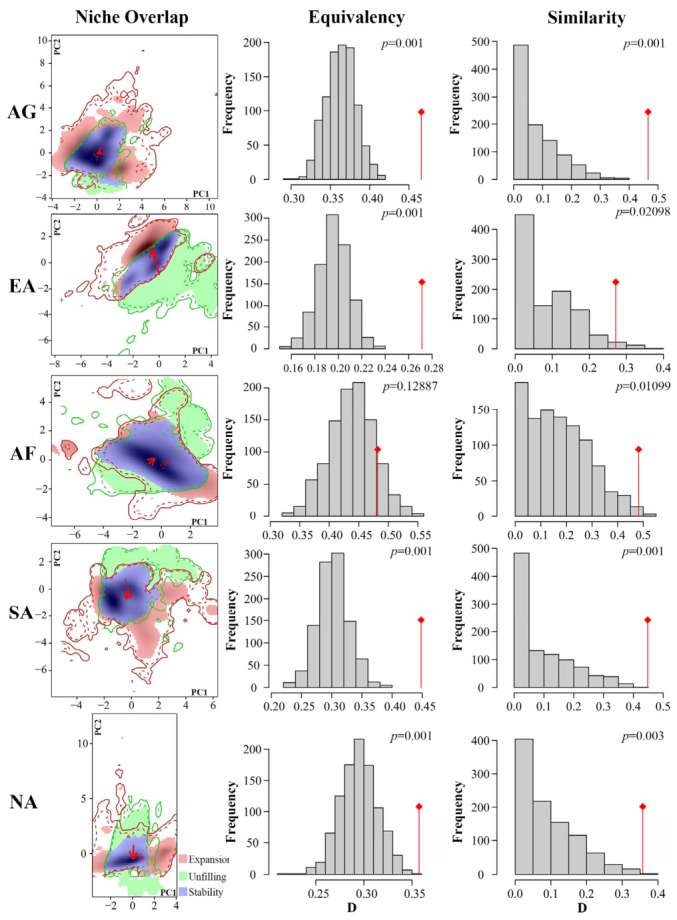
Comparison of climatic niches between the original habitat (Australia) and the invasive site. AG, EA, AF, SA and NA represent all globally invasive regions, Eurasian continent, Africa, South America and North America. Niche overlap plot. The green solid and dashed lines, respectively, represented the 100% and 75% background environments of the native range. The red solid and dashed lines, respectively, represented the 100% and 75% background environments of the invasive ranges. The solid arrow indicated the shift in the ecological niche centroid from the origin to the invasion site. The dashed arrow indicated the environmental spatial transfer from the origin to the invasion site. Niche equivalence and similarity tests between the native and invasive regions of *P. recurva*, with diamonds representing observed Schoener’s *D* values.

COUE framework analysis (Table 1) revealed high niche stability (0.718) and moderate niche overlap (0.466) across the global invaded range, alongside relatively pronounced niche expansion (0.282). Niche comparisons between native and invaded ranges disclosed heterogeneous shifts across continents (Figure 8). Schoener’s *D* values for South America (0.448) and Africa (0.482) indicated moderate overlap; North America (D = 0.357) and Eurasia (D = 0.271) displayed lower overlap. With the exception of Africa, where niche expansion was minimal (0.090), all other invaded regions exhibited more pronounced niche expansion (>0.1). Populations in Eurasia and North America also displayed high niche unfilling (0.549 and 0.406, respectively).

Niche equivalency and similarity tests between native (Australian) and invaded ranges yielded significant results (*p* < 0.05) across all regions for similarity and across all regions except Africa for equivalency (Figure 8). Significant equivalency test results for non-African ranges indicated that those invaded-range niches differed from the native-range niche, whereas significant similarity tests confirmed that climatic conditions remained comparable between Australia and all invaded ranges. The non-significant equivalency test for Africa suggested potential niche equivalence between African and Australian populations, while the significant similarity test nonetheless indicated greater niche similarity than expected by chance.

**Figure 9:**
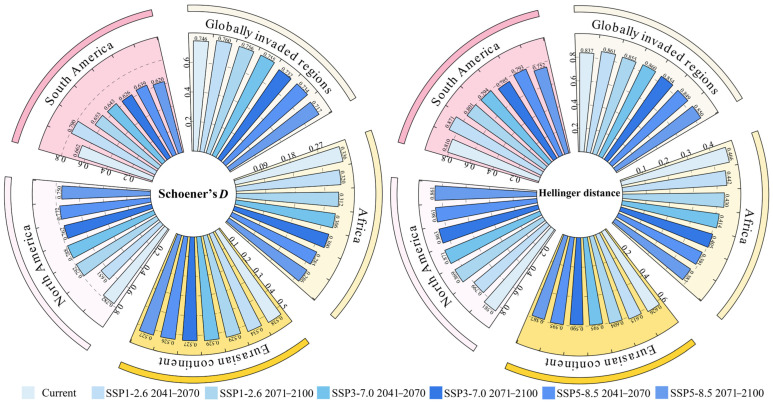
The Schoener’s *D* and Hellinger distance between the native and invaded ranges of *P. recurva* under different prediction results.

The niche overlap between native- and invaded-range potential distribution models was quantified using Schoener’s *D* and modified Hellinger distance I. Eurasia, South America, North America, and the global invaded range collectively exhibited moderate to high niche overlap (D > 0.526, I > 0.587; Figure 9). In Africa, niche overlap was comparatively low and decreased progressively across climate scenarios.

## 4. Discussion

Native to Australia, *Phoracantha recurva* has established populations throughout North America, South America, Europe, and Africa. Its recent detection in the Azores Islands within the Macaronesian region [38] further demonstrates the ability of invasive organisms to overcome biogeographical barriers and expand their ranges through multiple introduction pathways. Identifying the potential distribution of such invasive species is therefore essential for developing effective prevention, surveillance, and source-control strategies.

### 4.1. Model Prediction and Key Pest Control Regions

The optimized MaxEnt model provided robust predictions of the potential distribution of *P. recurva*. Both AUC values and the Boyce index approached or exceeded 0.9 (Figure 2 and Figure S5), indicating excellent predictive performance and close agreement between model outputs and observed occurrence records. Highly and moderately suitable habitats were concentrated primarily in southeastern South America, the Indochinese Peninsula, and the Indian Peninsula (Figure 3). Brazil, the world’s largest producer of eucalyptus, allocates approximately 76% of its planted forest area to *Eucalyptus* spp. [39]. However, extensive monoculture plantations often intensify pest outbreaks, particularly those caused by Australian invasive insects and native herbivores [40,41]. Most Brazilian eucalyptus plantations are concentrated in Minas Gerais (26%), São Paulo (13%), and Mato Grosso do Sul (15%) [42]. Because these states were also predicted as highly suitable for *P. recurva*, stabilizing populations may be present in these regions. Consequently, local plantations may face a dual invasion risk arising from both potential domestic source populations and transboundary introductions.

China also supports a vast eucalyptus industry. According to the Ninth National Forest Resources Inventory, Guangxi, Guangdong, Yunnan, Fujian, and Sichuan rank among the five provinces with the largest eucalyptus plantation areas [43,44]. Within Yunnan Province, counties such as Shiping, Jianshui, and Kaiyuan fall within moderately to highly suitable areas, as do Nanjing and Yunxiao counties in Fujian Province. Given the extensive international trade in eucalyptus products and planting materials, quarantine agencies should strengthen inspection and surveillance efforts, particularly by intensifying analyses of interception records, to prevent further introduction and spread of *P. recurva*.

The successful biological control of *P. semipunctata* in California using the egg parasitoid *Avetianella longoi* [45] provides a useful management precedent. Nevertheless, *P. recurva* exhibits effective defensive responses against this parasitoid [46], potentially limiting its efficacy. Therefore, in areas where both *Phoracantha* species coexist, alternative management approaches, including genetic improvement and intraspecific hybridization aimed at enhancing host resistance, may be required to ensure sustainable plantation management.

Despite the high reliability of the model predictions presented in this study, two methodological limitations warrant explicit acknowledgment. First, although spatial filtering was performed using spThin to mitigate biases induced by spatial autocorrelation and clustered occurrence records, this approach primarily addresses oversampling in data-rich regions and cannot fully compensate for the complete absence of field survey data in other potentially suitable habitats. This may result in a conservative estimation of the pest’s potential distribution range [47,48]. Second, the classification of continuous MaxEnt outputs into distinct habitat suitability classes remains an ongoing topic of debate, and no universally accepted grading standard has been established to date [49]. The Jenks Natural Breaks method was adopted in this study for its strength in enabling data-driven classification based on the inherent distribution characteristics of the suitability surface, without the need for arbitrarily defined fixed thresholds. However, its core limitation is that these statistically optimal breakpoints do not necessarily correspond to biologically meaningful inflection points in habitat quality, which complicates the interpretation of spatial habitat patterns and hinders the development of a standardized framework for delineating suitable areas across multiple species.

### 4.2. Impact of Climate Change on the Distribution of P. recurva

Jackknife analysis identified temperature as the primary determinant of *P. recurva* distribution, with the minimum temperature of the coldest month (BIO6) contributing the greatest amount of unique information (Figure 7A). Previous field studies have shown that *P. recurva* remains active for prolonged periods during cool seasons and can exploit transient winter warming events for oviposition [50,51]. Favorable winter temperatures therefore appear to create the thermal window this species requires for reproduction and population persistence.

Climate change alters species distributions by reshaping regional temperature and precipitation regimes, thereby driving range expansions, contractions, and shifts. GCMs represent a major source of uncertainty in predicting species’ potential geographic distributions under climate change, and their contribution to this uncertainty increases over longer projection periods. To account for this, we employed the ensemble mean of multiple global climate models for our simulations [52]. Under future climate scenarios, the total suitable area for *P. recurva* was projected to decline overall. Nevertheless, suitable habitats expanded poleward in several regions. Under the SSP3-7.0 scenario, for instance, the latitude of the northernmost grid cell at the distribution boundary reached approximately 74.49° N during 2041–2070 and further shifted to 80.15° N during 2071–2100 (Figure 5). These results are consistent with previous studies documenting climate-driven poleward range shifts. Chen et al. [53] documented poleward and upslope range shifts across multiple taxa under climate change, with migration rates accelerating as warming intensifies. Similarly, Hickling et al. [54] analyzed distributional changes in 359 species spanning 16 taxonomic groups in Britain and found a prevailing pattern of northward migration and upward elevational shifts during warming periods. Our results lend further support to these general patterns.

Species distributions are shaped by multiple interacting factors beyond climate, including host availability, natural enemies, human activity, and intrinsic ecological traits, any of which can constrain colonization success. Where climatic limits on the host plant are more restrictive than those on the target species, overall adaptability may be compromised [55]. Integrating biotic interactions, habitat patchiness, and anthropogenic disturbance into future modeling frameworks—alongside the development of comprehensive risk assessment protocols that address the full invasion trajectory—represents a priority for subsequent research.

### 4.3. Niche Dynamics Analysis

The niche comprises the suite of biotic and abiotic conditions under which a species can persist and maintain stable population sizes [55,56]. We employed two complementary approaches—analysis in the observed environmental space and in the potential geographical distribution—to compare niches between the native and invaded ranges [57].

Niche conservatism manifests through high niche stability and significant niche similarity [58]. Within the observed environmental space, despite a degree of niche expansion exceeding 0.1 across the global invaded range, *P. recurva* retained a high stability coefficient (0.718) and a low unfilling proportion (0.043). Niche similarity exceeded random expectations. Overall, the species has conserved its native niche characteristics throughout its global invasion. Continental-scale comparisons, however, reveal heterogeneity. During invasion of Eurasia, niche unfilling was comparatively large (0.549). The vast territory of the Eurasian continent encompasses varied topography, broad climatic gradients, and nearly all vegetation types [59]; *P. recurva* has therefore not yet fully exploited the available environmental space and retains substantial invasion potential there. Short residence time, dispersal barriers, biotic interaction constraints, and random genotype loss through genetic drift may all contribute to this unfilling [30,60].

Under the combined influence of climatic change and biotic interactions, the magnitude of niche differentiation is not static [60]. Comparing niche overlap between native and introduced ranges as projected by MaxEnt revealed increased overlap in all regions except Africa, suggesting that *P. recurva* is progressively occupying environmental conditions resembling those of its native Australian range. Although the African population currently exhibits niche equivalency with the native population and shows a relatively limited degree of niche expansion, the climatic conditions in this region differ substantially from those in Australia. Therefore, *P. recurva* may not yet have fully dispersed into other potentially suitable areas. As the invasion process advances, further niche differentiation may occur. We also note that the occurrence records in Africa are relatively limited. Liu et al. [32] reported that the number of introduction occurrences did not significantly affect niche expansion in terrestrial ectotherms. However, methods relying on kernel-smoothed density estimates may be strongly affected by differences in sampling intensity [61]. Therefore, for regions exhibiting similar conditions, future studies should consider integrating multiple methods for assessing niche dynamics, thereby allowing the results to provide complementary insights. 

Liu et al. [62] reported the occurrence of *P. semipunctata* in Zhongshan, Dongguan, and Guangzhou in southern China. According to niche and coexistence theory, ecologically similar species often exploit comparable resources and occupy similar habitats [63]. Because *P. recurva* has not yet saturated its invaded niche space and because regions already colonized by its congener provide suitable environmental conditions, the likelihood of establishment in Asian eucalyptus plantations appears high. This risk is particularly pronounced in China, where extensive eucalyptus cultivation coincides with large areas predicted to be climatically suitable. Accordingly, proactive surveillance, strengthened quarantine measures, and early-warning systems should be prioritized in these high-risk regions.

## 5. Conclusions

We evaluated the global climatic suitability and niche dynamics of *P. recurva* using species distribution modeling. Suitable climates were predicted across a broad latitudinal range (60° N–55° S), encompassing several major Eucalyptus-producing countries, including China, Brazil, and Uruguay. While these projections indicate considerable exposure to climatic risk, actual invasion potential depends on additional factors such as propagule pressure, dispersal pathways, international trade routes, biological interactions, and host availability, which were not explicitly incorporated into this modeling framework. Climate change is projected to reduce the overall extent of climatically suitable habitat, with potential distributions in China and Europe shifting toward higher latitudes. *Phoracantha recurva* has largely retained its native climatic niche during invasion, yet extensive unoccupied niche space in Eurasia suggests that the species could expand into additional climatically suitable areas if introduction and establishment conditions are met. Conversely, declining niche overlap among African populations suggests that niche dynamics may continue to evolve during the invasion process. Collectively, these findings underscore the value of combining climatic suitability projections with targeted surveillance and quarantine measures to mitigate the risk of *P. recurva* establishment in vulnerable Eucalyptus plantations worldwide.

## Figures and Tables

**Table 1:** Climatic niche differences between the native and invaded ranges of *P. recurva*.

Invasive Regions	Schoener’s *D*	Expansion	Stability	Unfilling
Globally invaded regions	0.466	0.282	0.718	0.043
Eurasian continent	0.271	0.369	0.631	0.549
North America	0.357	0.333	0.667	0.406
South America	0.448	0.229	0.771	0.198
Africa	0.482	0.090	0.910	0.181

## Data Availability

The data is included in the article. For the data provided in this study, see the Section 2.1 and Section 2.2 in the text.

## Supplementary Materials

The following supporting information can be downloaded at https://www.mdpi.com/article/10.3390/insects17070729/s1, Table S1. 19 bioclimatic factors; Table S2. Environmental variables and contribution rate of *P. recurva* regional model; Figure S1. Pearson correlation analysis of climatic factors; Figure S2. Main parameter settings of the MaxEnt model. (A) Environmental variables and their contribution rates for predicting the potential geographical distribution of *P. recurva*. (B) The FC and RM of the MaxEnt model; Figure S3. Correlation Analysis of environmental variables in the regional model of *P. recurva*. (A) Eurasian continent, (B) Africa, (C) North America, (D) South America; Figure S4. The FC and RM of the regional model. (A) Eurasian continent, (B) Africa, (C) North America, (D) South America; Figure S5. Accuracy evaluation of the regional model for *P. recurva*. (A,B) Boyce index and RUC curves of the Eurasian Continental regional model; (C,D) Boyce index and RUC curve of African regional model; (E,F) Boyce index and RUC curve of the North American Continental regional model; (G,H) Boyce index and RUC curve of South American Continental regional model; Figure S6. Variations in suitable area and omission rate along cumulative thresholds of the global MaxEnt model; Figure S7. Variations in suitable area and omission rate along cumulative thresholds of MaxEnt models for four continents: (A) Eurasian continent, (B) Africa, (C) North America, (D) South America; Figure S8 The predicted geographical distribution of *P. recurva* under near future climate conditions; Figure S9. Changes in the suitable range of *P. recurva*.
