# Supplementary material for: Global Invasion Potential and Niche Dynamics of Phoracantha recurva Newman, 1840 (Coleoptera: Cerambycidae) Under Climate Change
Source: Insects. 2026 Jul 15;17(7):729. doi: 10.3390/insects17070729 (PMC13410602; doi:10.3390/insects17070729)
Supplement: Supplementary file 1 [file insects-17-00729-s001.zip › insects-4388502-supplementary.pdf]

Table S1. 19 bioclimatic factors

Climate variables		Unit
BIO1	Annual Mean Temperature	℃
BIO2	Mean Diurnal Range	℃
BIO3	Isothermality (×100)	-
BIO4	Temperature Seasonality	-
BIO5	Maximum Temperature of Warmest Month	℃
BIO6	Minimum Temperature of Coldest Month	℃
BIO7	Temperature Annual Range	℃
BIO8	Mean Temperature of Wettest Quarter	℃
BIO9	Mean Temperature of Driest Quarter	℃
BIO10	Mean Temperature of Warmest Quarter	℃
BIO11	Mean Temperature of Coldest Quarter	℃
BIO12	Annual Precipitation	mm
BIO13	Precipitation of Wettest Month	mm
BIO14	Precipitation of Driest Month	mm
BIO15	Precipitation Seasonality	-
BIO16	Precipitation of Wettest Quarter	mm
BIO17	Precipitation of Driest Quarter	mm
BIO18	Precipitation of Warmest Quarter	mm
BIO19	Precipitation of Coldest Quarter	mm

Table S2 Environmental variables and contribution rate of *P. recurva* regional model

	Eurasia	Africa	Northern America	Southern America
BIO1	-	0.3	0.065	-
BIO2	0.044	0.039	0.021	0.124
BIO3	-	0.015	0.41	0.119
BIO4	-	0.126	-	-
BIO5	-	-	-	-
BIO6	0.198	-	0.017	-
BIO7	-	-	-	0.015
BIO8	-	0.061	0.033	-
BIO9	0.107	-	-	-
BIO10	-	-	-	-
BIO11	-	0.122	-	0.535
BIO12	-	-	-	-
BIO13	-	-	-	0.058
BIO14	-	0.158	0.32	-
BIO15	0.026	-	0.131	0.028
BIO16	-	-	0.012	-
BIO17	-	-	-	-
BIO18	0.227	0.038	-	0.091
BIO19	0.398	0.141	-	0.03

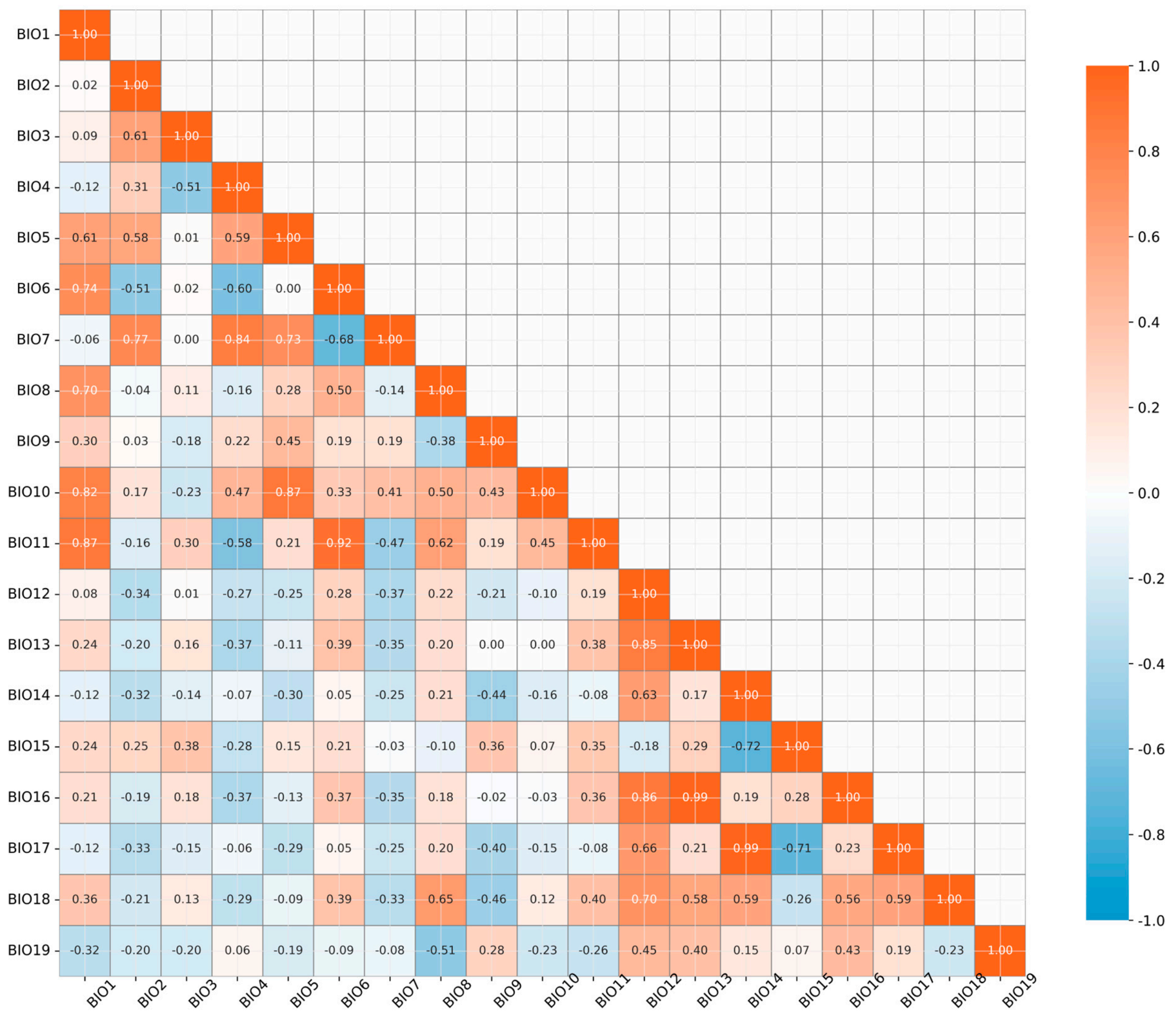

Figure S1. Pearson correlation analysis of climatic factors.

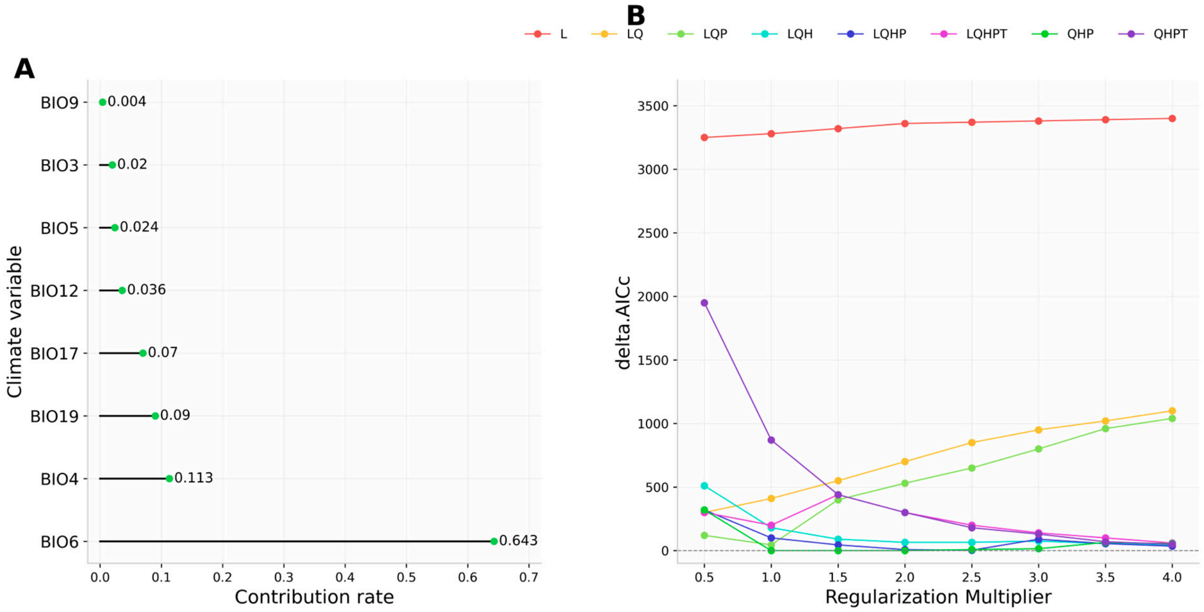

Figure S2. Main parameter settings of the MaxEnt model. (A) Environmental variables and their contribution rates for predicting the potential geographical distribution of *P. recurva*. (B) The FC and RM of the MaxEnt model.

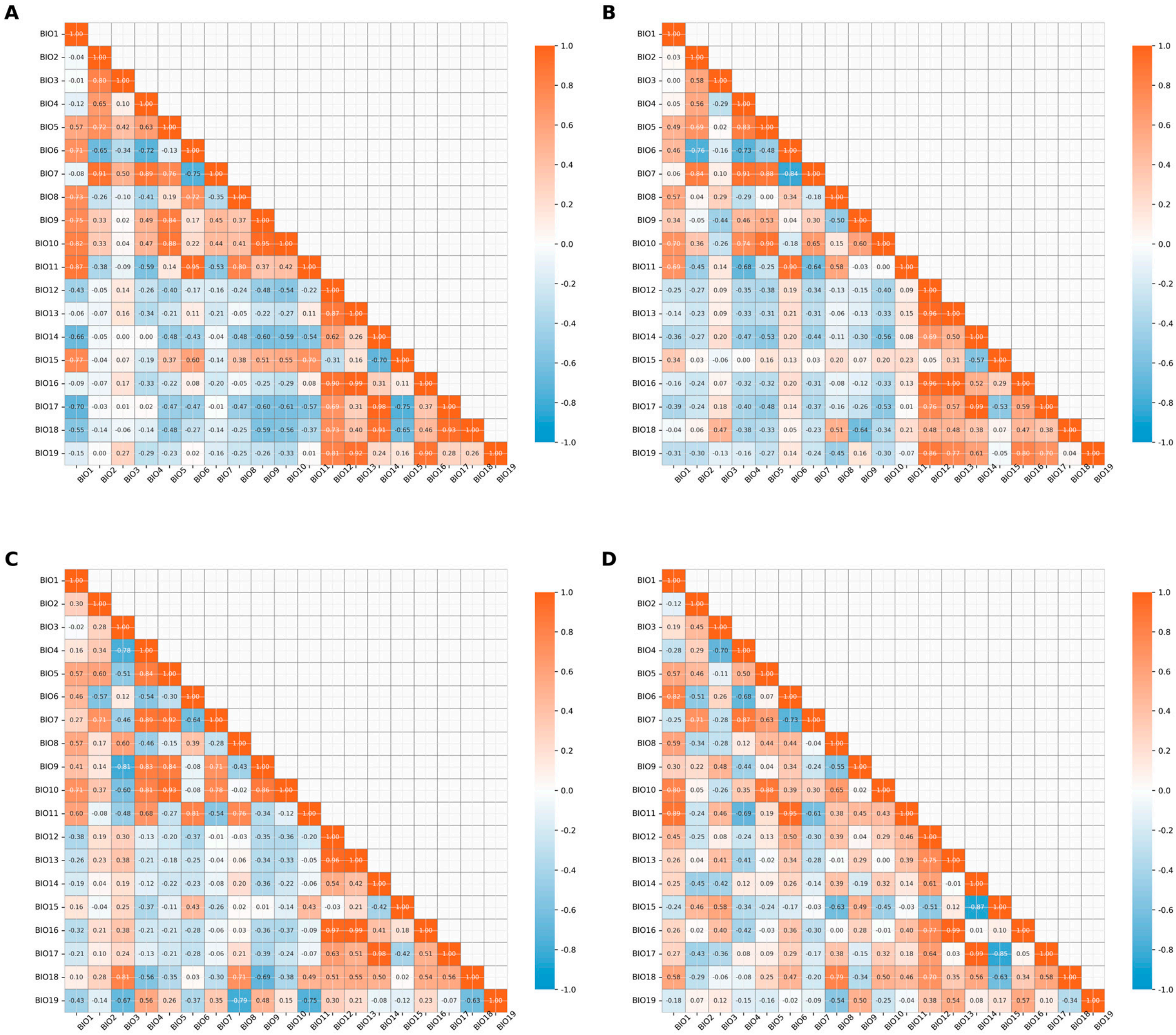

Figure S3. Correlation Analysis of environmental variables in the regional model of *P. recurva*. (A) Eurasian continent, (B) Africa, (C) North America, (D) South America.

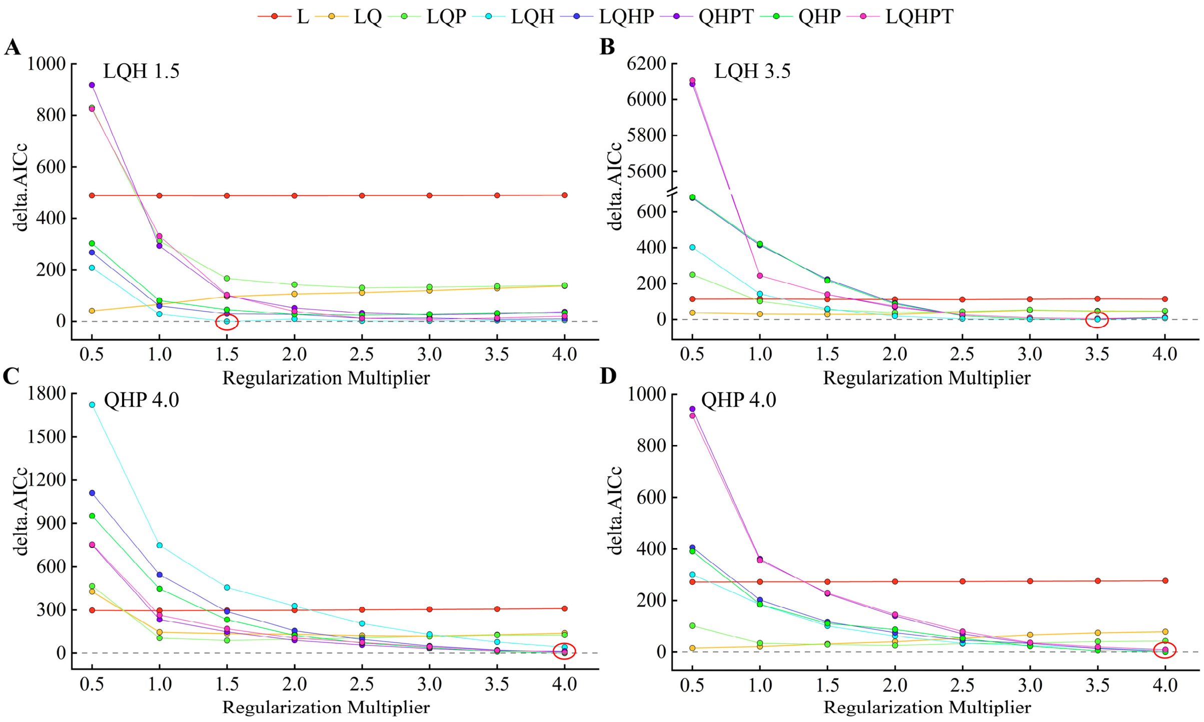

Figure S4. The FC and RM of the regional model. (A) Eurasian continent, (B) Africa, (C) North America, (D) South America.

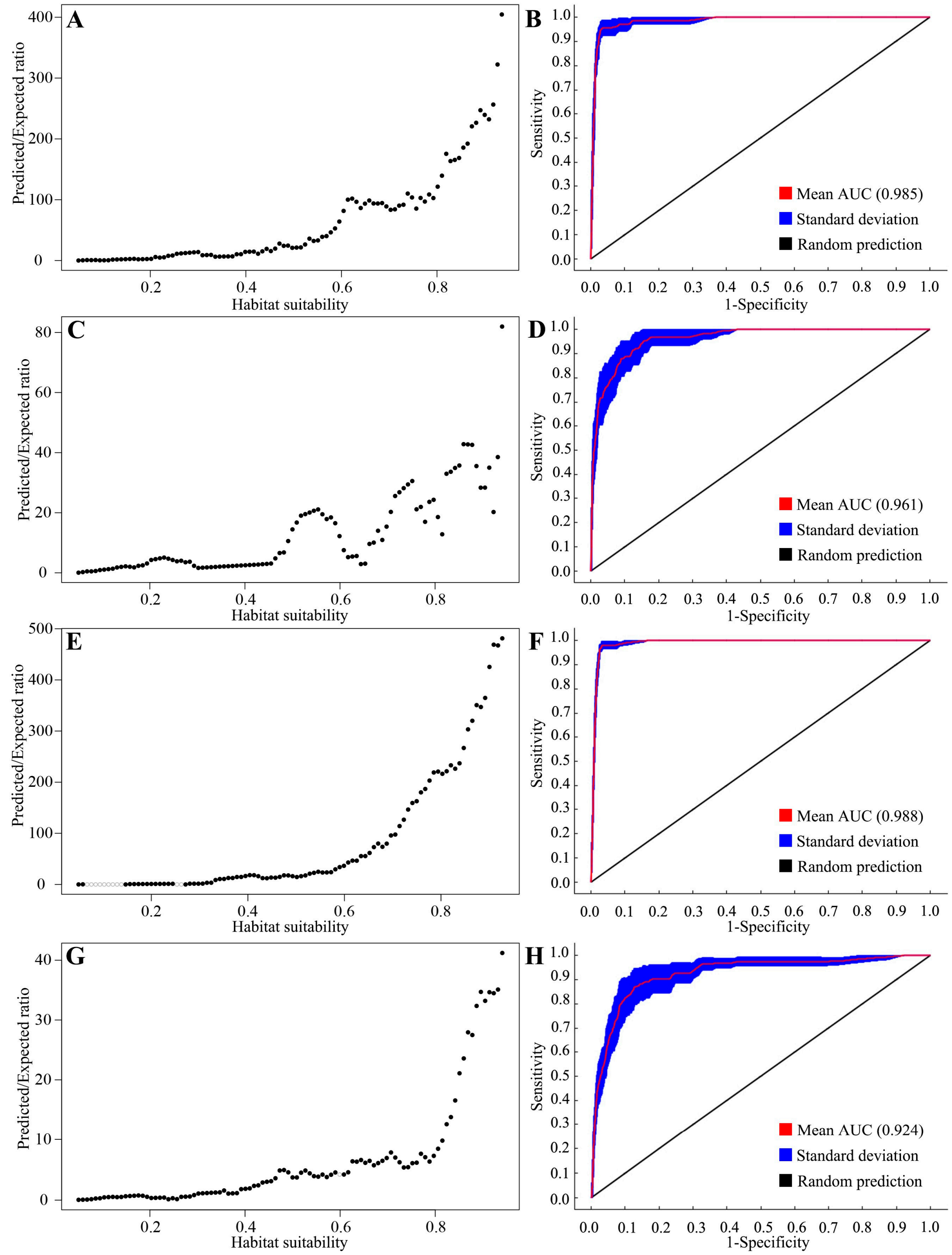

Figure S5. Accuracy evaluation of the regional model for *P. recurva*. (A, B) Boyce index and RUC curves of the Eurasian Continental regional model; (C, D) Boyce index and RUC curve of African regional model; (E, F) Boyce index and RUC curve of the North American Continental regional model; (G, H) Boyce index and RUC curve of South American Continental regional model.

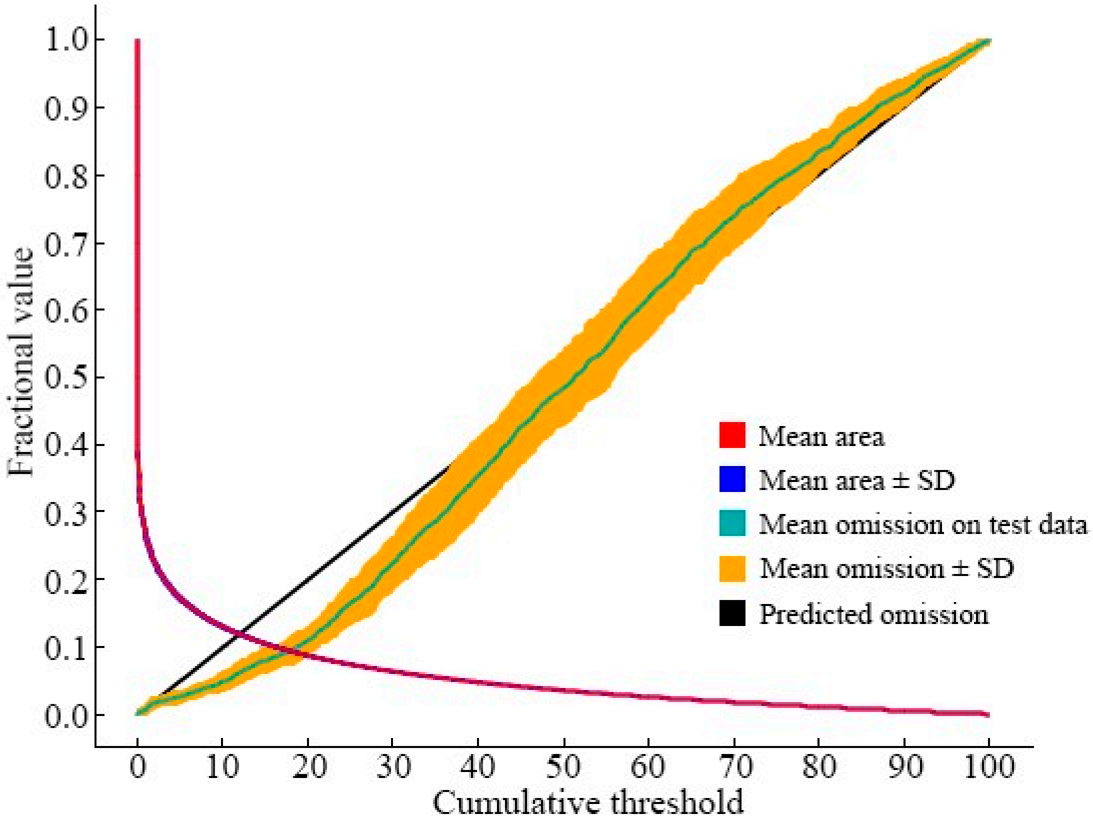

Figure S6. Variations in suitable area and omission rate along cumulative thresholds of the global MaxEnt model.

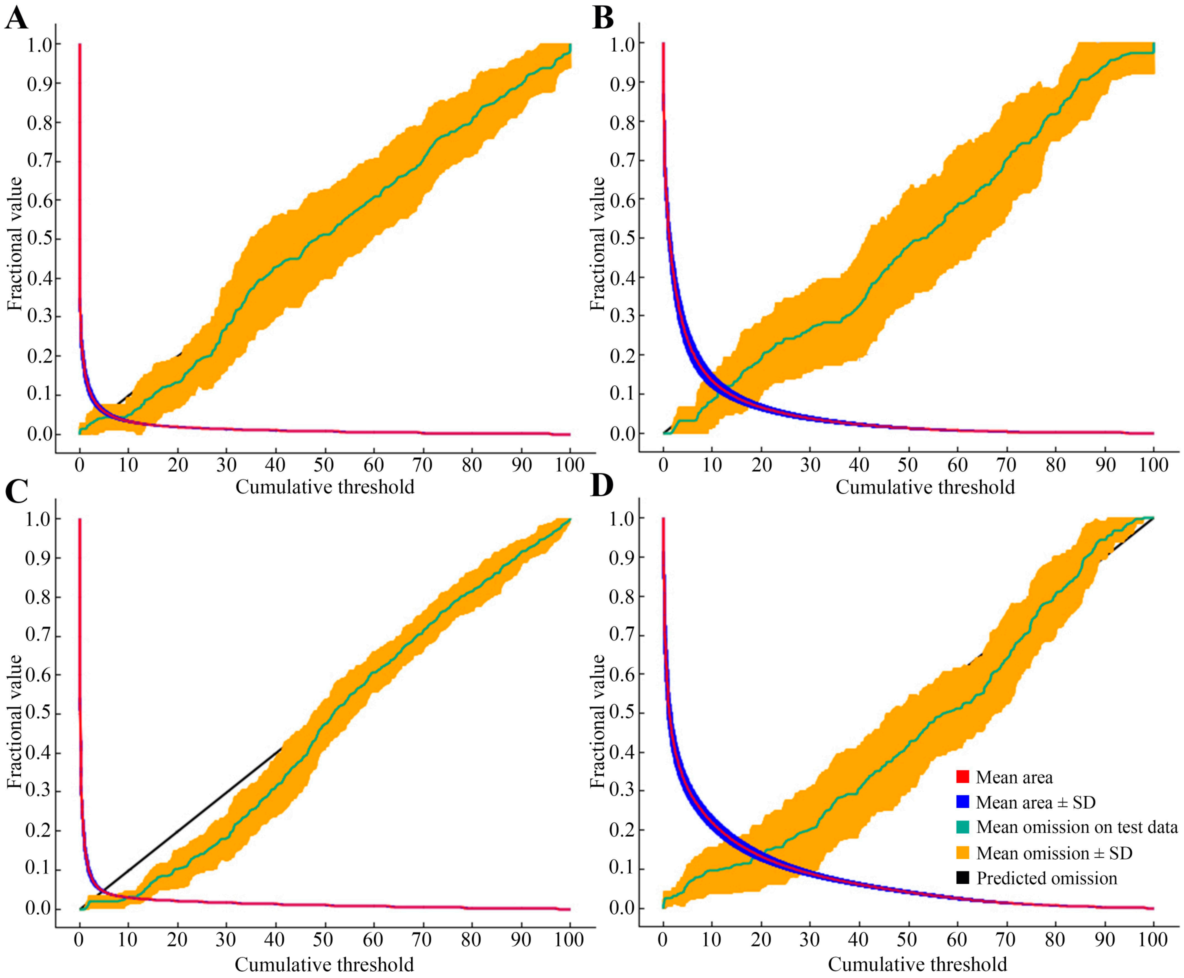

Figure S7. Variations in suitable area and omission rate along cumulative thresholds of MaxEnt models for four continents: (A) Eurasian continent, (B) Africa, (C) North America, (D) South America.

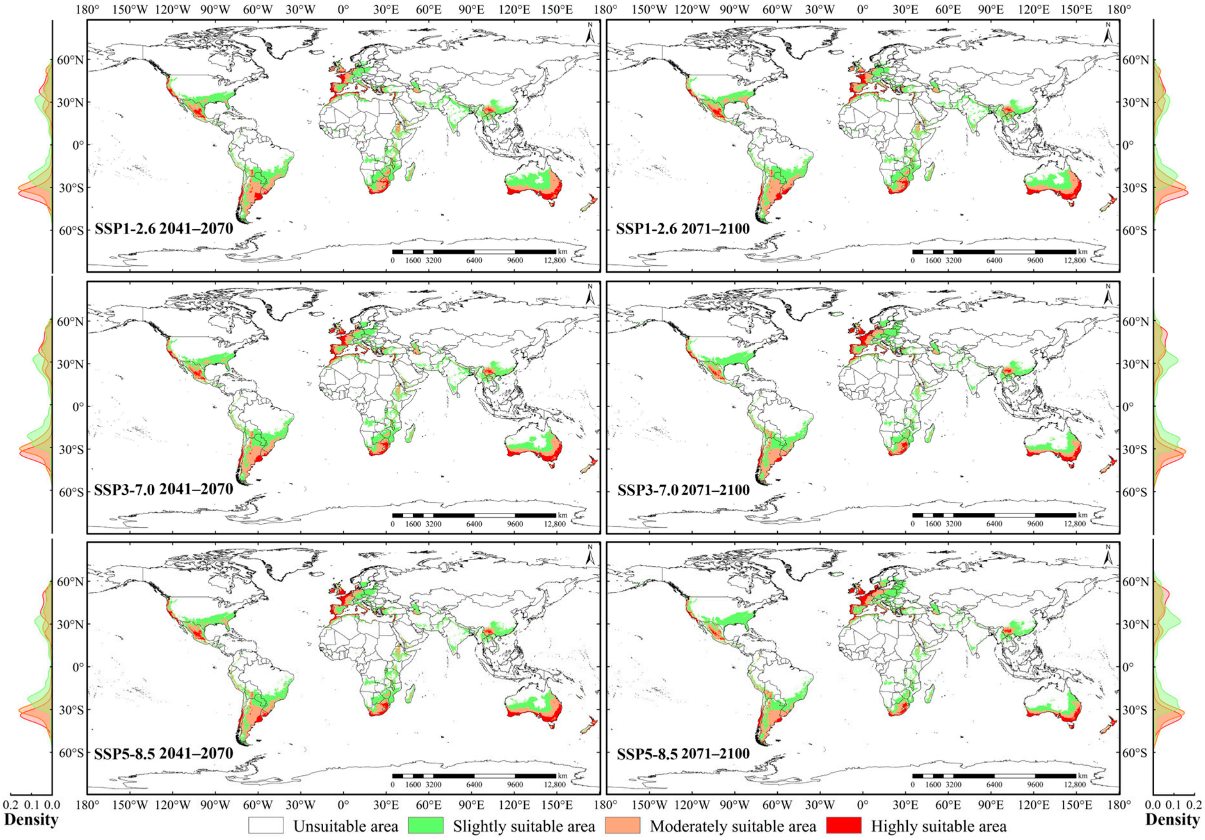

Figure S8 The predicted geographical distribution of *P. recurva* under near future climate conditions.

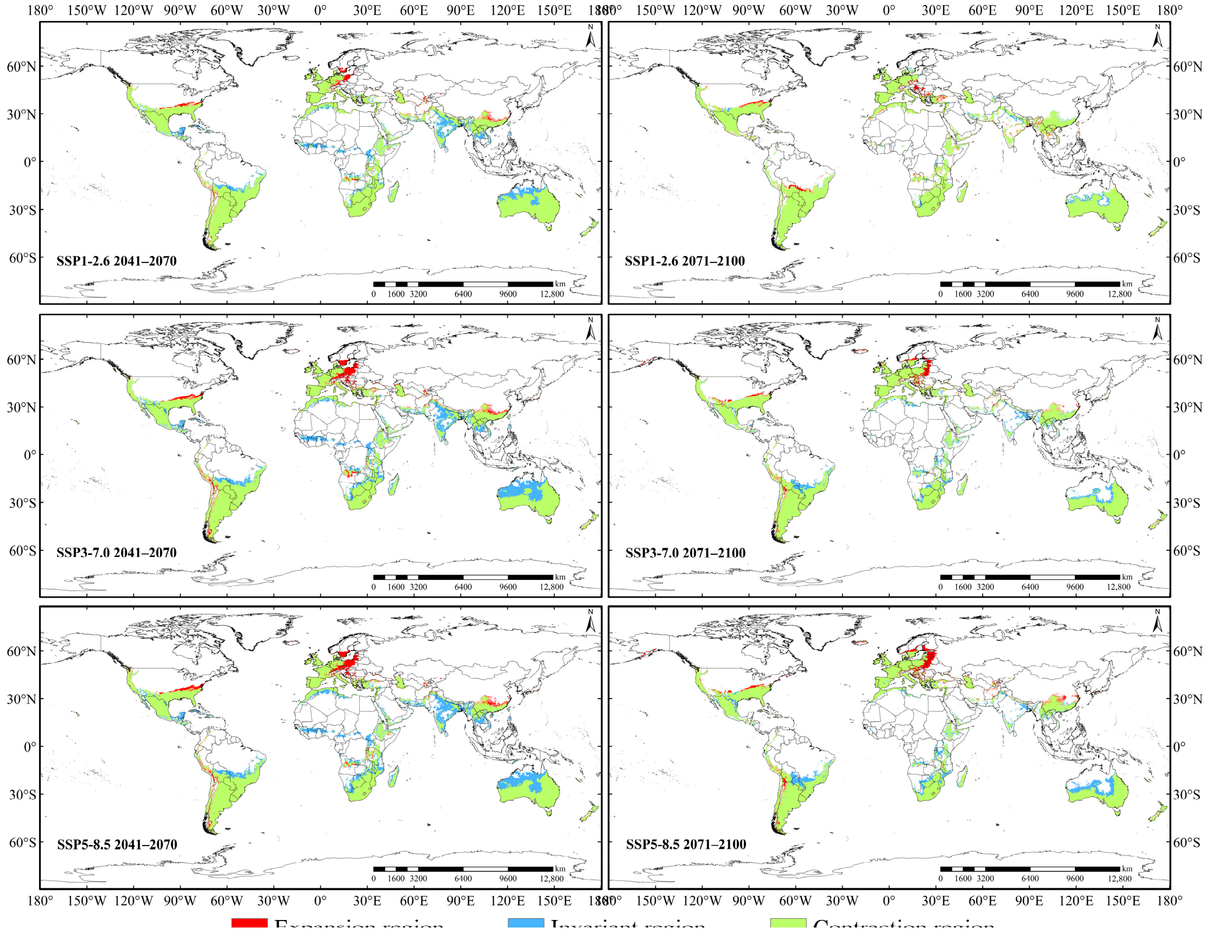

Figure S9. Changes in the suitable range of *P. recurva*.
